# Ameliorative effects of green tea extract from tannase digests on house dust mite antigen-induced atopic dermatitis-like lesions in NC/Nga mice

**DOI:** 10.1007/s00403-018-01886-6

**Published:** 2019-01-07

**Authors:** YeonSil Hwang, BoYoon Chang, TaeYoung Kim, SungYeon Kim

**Affiliations:** 1 Institute of Pharmaceutical Research and Development, College of Pharmacy, Wonkwang University, Iksan, Jeonbuk 54538 South Korea; 2 #703, Technology Development Center, BTC Corporation, 705 Haean-ro, Sangnok-gu, Ansan, Gyeonggi-do 15588 Republic of Korea

**Keywords:** Atopic dermatitis (AD), Green tea extract, Tannase, House dust mite, NC/Nga

## Abstract

Atopic dermatitis (AD) is one of the most common chronic inflammatory skin diseases, which is affected by several factors. Anti-histamines, steroids, and immunosuppressive agents have been used for the treatment of AD. However, many studies have reported that long-term use and abuse of these drugs causes many side effects. This study was performed to evaluate the ameliorative effect of green tea extract on AD-like lesions in NC/Nga mice. Green tea extract from tannase digest (GTT), beta-hexosaminidase, and histamine were measured in IgE-antigen complex-stimulated RBL-2H3 cells. Dorsal skin application of house dust mite-ointment induced AD-like symptoms in NC/Nga mice. Dermatitis scores, skin moisture, transepidermal waterloss (TEWL), thickness of skin and ear, T-cell proliferation, levels of immunoglobulins and cytokines, and infiltration of mast cell were measured to assess the degree of AD induction. Skin moisture and TEWL were measured using probes, and ELISA was performed to measure the immunoglobulin and cytokine levels in blood. GTT was selected based on its ability to inhibit the release of beta-hexosaminidase and histamine in IgE-antigen complex-stimulated RBL-2H3 cells. Oral administration of GTT significantly suppressed the skin inflammation and symptoms of AD-like skin lesions in NC/Nga mice. GTT may have a potential therapeutic effect in the treatment of AD.

## Introduction

The skin plays an important role in the body defense against harmful stimuli. Atopic dermatitis (AD) is a chronic inflammatory skin disease that causes severe itching and dry skin after contact with aeroallergens such as house dust mites, pollen, and animals [35]. Atopic dermatitis breaks down the barrier function of skin epidermis, causing external antigens to enter the skin and cause allergic reactions in the entire body. It mainly affects young children, but 25% of the individuals with AD continue to suffer from it until adulthood [23]. Moreover, its prevalence has increased in urbanized societies over recent decades [39].

The treatment of AD involves the use of steroids, anti-histamines, and immunosuppressive agents [30]. Specifically, corticosteroids and calcineurin inhibitors are useful early stage treatments that can rapidly alleviate AD symptoms, but both show side effects such as steroid phobia, paradoxical skin disease, and skin irritation [10]. In addition, many studies have reported that long-term use or even abuse of these agents may result in various side effects, so recent studies have focused on complementary therapies based on alternative medicine. Several such studies have shown that natural products with anti-inflammatory properties have the potential to treat skin inflammatory disorders such as AD.

It was reported that 70% ethanol extract of *Artemisia capillaris* exerted inhibitory effects on AD-like skin lesions via the downregulation of serum histamine content and IgE expression [11]. Perilla leaf extract may inhibit the symptoms of AD via the suppression of macrophages by tumor necrosis factor-α (TNF-α). In this case, the main active ingredients are polyphenols, including rosmarinic acid, luteolin, and apigenin [33].

Green tea contains various active components, and it has been widely used as a herbal medicine. The bioactive compounds of green tea include polyphenols, polysaccharides, amino acids, and vitamins [8]. Catechins account for 75–80% of soluble ingredients in tea. The four types of tea catechins include epicatechin (EC), epigallocatechin (EGC), epicatechin gallate (EGC), and epigallocatechin gallate (EGCG). Tea catechins have received increased research interest because of their positive physiological and pharmacological effects combined with antimutagenic, anticarcinogenic, and antitumorigenic activities [3, 8]. High concentration enhances taste intensity, but also results in decreased taste palatability. Catechin bioavailability is very poor because of its large molecular size and the number of hydrogen bonds. Many enzymes have been used for tea processing and quality improvement for decades [3, 14]. Specifically, Tannase treatment is one of the best solutions to the drawbacks mentioned above. Tannase treatment of green tea extract is an efficient method for the biotransformation of catechins with enhanced radical-scavenging activity [4].

Recently, various studies suggested a beneficial effect of green tea on several dermatological disorders such as UV-induced inflammatory reaction [9]. In addition, bath therapy with green tea extracts is an effective treatment for AD [18]. However, it is not known whether oral administration of green tea extract or green tea extract from tannase digests can affect the AD-like symptoms in NC/Nga mice.

Thus, the present study aimed to select an effective green tea extract from tannase digests using in vitro mast cell degranulation assay and evaluate its effect in reducing AD-like symptoms in house dust mite antigen-induced mouse model.

## Methods

### Extraction a of green tea extract from tannase digests (GTT)

The green tea extract from tannase digests (GTT) used in the experiments was provided by BTC. The following are the manufacturing process. For the extraction, 1 kg ground tea leaves (Osullocfarm, Gangjin, Korea) were added with 15 L water in a 50 L vessel (COSMOS660, Kyungseo E&P., Korea), and then, it was extracted at 90 °C for 6 h. After the extracts were filtered (5 µm filter) to remove the insoluble components, 4 g tannase was added and the mixture was incubated at 40 °C for 2 h under pH 5.0–5.5. Following this, to inactivate the enzymes, the enzymatic reaction was stopped by heating the mixture at 90 °C for 1 h. The product was concentrated to 40 brix at 60 °C in a vacuum evaporator and dried in a freeze-dryer (FD8505, Ilshin Corp., Korea) to yield GTT (47.5%, w/w). The content of the marker galic acid, EGC, Caffeine, and EC in GTT was quantitated using high-performance liquid chromatography (HPLC).

### HPLC estimation of green tea extract from tannase digests (GTT)

**Fig. 1:**
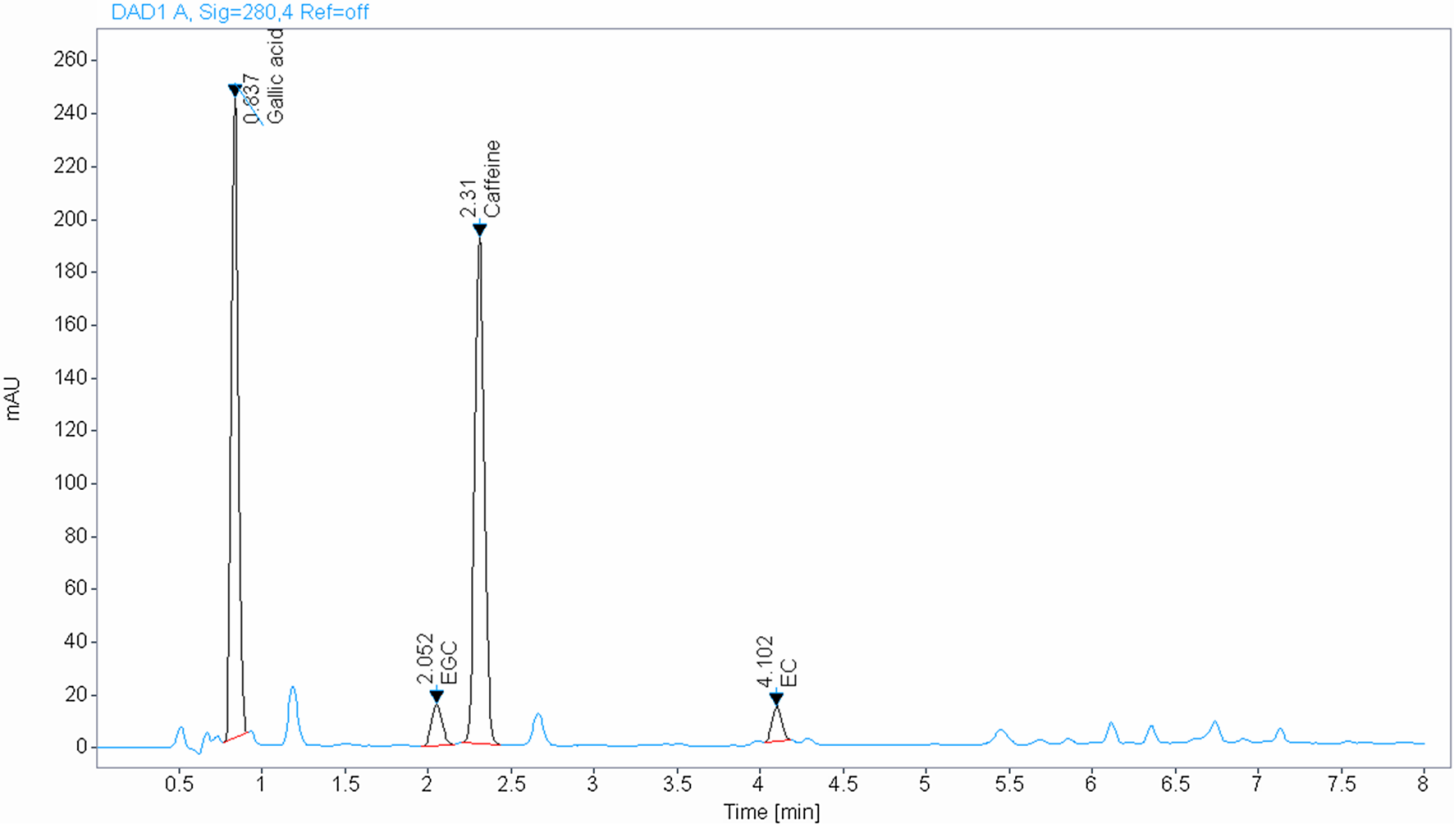
HPLC analysis of green tea extract from tannase digest (GTT)

For HPLC analysis, green tea extract from tannase digests (GTT) was dissolved in HPLC-grade methanol to a concentration of 5 mg/mL and filtered through a 0.45 µm syringe filter. The reference standard comprised 0.5 mg gallic acid, caffeine, EGC, and EGCG in 1 mL HPLC-grade methanol. The HPLC system consisted of a model 515 pump and model 717 autosampler (Waters, Beverly, MA, USA). Reverse-phase separation was performed at room temperature using a Agilent Poroshell EC-C18 (I.D 4.6 × 50, 2.7 µm). The mobile phase included water containing 0.1% H_3_PO_4_ and acetonitrile. The flow rate was maintained at 1 mL/min and the peak was detected at 280 nm. The marker compound was identified by comparing the UV spectrum and retention time (Fig. 1).

### Anti-oxidant activities of green tea extracts

To confirm the anti-oxidant activity of green tea extracts, 2,2-diphenyl-1-picrylhydrazyl (DPPH) radical-scavenging assay was performed using the method described by Klouwen [20]. The extracts were added with 0.2 mM DPPH and incubated for 30 min. Absorbance was measured at 520 nm. The inhibition rate was calculated as follows: (1 − *A*/*B*) × 100, where *A* is the absorbance of the sample and *B* is the absorbance of the control. Vitamin C (50 µM) was used as a reference. Superoxide-radical-scavenging assay was also performed using the SOD assay kit-WST (Dojindo, Tokyo, Japan) according to the manufacturer’s instructions. Optical density was measured at 450 nm.

### Cell culture and cytotoxicity of green tea extracts on RBL-2H3 cells

RBL-2H3 cells were obtained from Korea Cell Line Bank (Seoul, Korea) and cultured in RPMI with 10% FBS and 1% antibiotics at 37 °C in a humidified incubator under 5% CO_2_. Cells were plated at a density of 2 × 10^4^ cells/well in a 96-well microplate and incubated for 24 h. The cells were then cultured with green tea extracts at different concentrations (10–100 µg/mL). After 24 h incubation, the medium was changed, and 1 mg/mL methylthiazol tetrazolium (MTT, Sigma-Aldrich, St. Louis, MO, USA) was added, followed by the incubation of cells for another 2 h at 37 °C. The precipitate was dissolved in DMSO and the absorbance was measured at 540 nm.

### Measurement of β-hexosaminidase and histamine contents on RBL-2H3 cells

To measure the release of β-hexosaminidase and histamine on RBL-2H3 cells, cells were transferred onto a 24-well microplate (2 × 10^5^ cells/well), and incubated with 0.5 µg/mL anti-DNP-IgE overnight for cell sensitization. After washing with PIPES buffer (25 mM PIPES, 119 mM NaCl, 5 mM KCl, 5.6 mM glucose, 0.4 mM MgCl_2_, 1 mM CaCl_2_, 40 mM NaOH, and 0.1% BSA, pH 7.2), the cells were exposed to different concentrations of green tea extracts (10–100 µg/mL) or dexamethasone (1 µM) in PIPES for 1 h, and then stimulated with 50 µg/mL DNP-BSA for 24 h. The cell-culture supernatant was used for the measurement of β-hexosaminidase and histamine contents.

For β-hexosaminidase assay, 25 µL of cell-culture supernatant was incubated with 25 µL of 5 mM substrate solution (5 mM *p*-nitrophenyl-*N*-acetyl-*β*-d-glucosaminide dissolved in 0.2 M sodium citrate buffer, pH 4.5) at 37 °C for 2 h. The enzyme reaction was then terminated by adding 200 µL of stop solution (0.1 M Na_2_CO_3_/NaHCO_3_, pH 10.0) and the absorbance was measured at 405 nm using a microplate reader.

To measure the amount of histamine, 22.5 µL of 0.1 N HCl and 2.5 µL of 60% HClO_4_ were added to 25 µL of cell-culture supernatant and the mixed samples were centrifuged at 1500 rpm, 4 °C for 20 min. The supernatant was transferred to tubes containing 25 µL of 5 N NaOH, 150 µL of D.W, 500 µL of n-butanol, and 0.06 g of NaCl. Then, the tubes were centrifuged at 2000 rpm, 4 °C for 10 min. The upper organic phase was transferred to the new tubes, and added with 150 µL of 0.1 N HCl and 500 µL of n-heptane, followed by centrifugation at 2000 rpm, 4 °C for 10 min. To 100 µL of the lower aqueous phase, 200 µL of 1 N NaOH and 5 µL of reaction solution (1% o-phthaldialdehyde dissolved in methanol) were immediately added and mixed. After incubation at room temperature for 3 min (away from light), the reaction was terminated by the addition of 10 µL of 3 N HCl. The fluorescence intensity was measured for excitation at 360 nm and emission at 450 nm using a fluorometer (SPECTRAMAX M2, Molecular Devices Co. Ltd. USA).

### Induction of atopic dermatitis (AD) and oral administration of green tea extract from tannase digests (GTT)

Pathogen-free male NC/Nga mice, aged 6 weeks, with 22–25 g body weight, were purchased from the Central Lab Animal Incorporation (Seoul, Korea). The animals were housed at 23 ± 3 °C under a 12 h light/dark cycle and acclimatized for 7 days in laboratory condition before the experiments. Food and water were provided ad libitum. All animal protocols were approved by the Committee of Wonkwang University (protocol number: WKU17-112).

At the start of the experiment, mice were randomly divided into the following six groups (*n* = 7 for each group): normal, control, 3 mg/kg prednisolone, 100 mg/kg GTT, 200 mg/kg GTT, and 400 mg/kg GTT. The experimental unit was a cage with a single animal. Atopic dermatitis-like skin lesions were induced by topical application of Biostir-AD (Biostir, Kobe, Japan), a hydrophilic petrolatum-based ointment containing extract of house dust mite. The hair on the upper dorsal skin of mice was shaved and µL of 4% (w/v) sodium dodecyl sulfate was applied to the shaved skin for barrier disruption. After 30 min, Biostir-AD was applied to the area twice a week. D.W, prednisolone, and GTT were orally administrated daily for 4 weeks. On day 28, the mice were killed and the ear, dorsal skin, and blood were harvested for analysis. Any clinical signs related to toxicity, such as remarkable loss of body weight, were monitored in all groups of animals throughout the course of the experiment.

### Evaluation of dermatitis score and skin condition

The severity of dermatitis was evaluated weekly, according to the degree of five symptoms: erythema, dry skin, edema/excoriation, erosion, and lichenification, which were scored individually as 0 (none), 1 (mild), 2 (moderate), or 3 (severe). The sum of individual scores was taken as the dermatitis score. The skin condition was confirmed by the measurement of skin moisture, transepidermal waterloss (TEWL), and ear thickness. The thickness of mice ear was measured using vernier calipers (Mitutoyo Corp., Japan) weekly. An MPA corneometer, CM825 (Epigen, Seoul, Korea), was used for the measurement of skin moisture, and a tewameter TM300 probe (Epigen, Seoul, Korea) was applied for measurement of transepidermal waterloss. Skin moisture and TEWL data were analyzed using the associated application software. Measurements were done weekly for all the test subjects on the upper dorsal skin, under the same temperature and humidity.

### Determination of splenocyte proliferation

To determine ex-vivo cell proliferation, on day 28, we harvested the spleen from the mice and isolated the primary splenocytes. These primary splenocytes were seeded in a 96-well microplate at 5 × 10^5^ cells/well in complete RPMI. The cells were allowed to adhere for 4 h at 37 °C in a humidified 5% CO_2_ atmosphere. After 4 h of incubation, the cells were stimulated with lipopolysaccharide (LPS, 10 µg/mL) or concanavalin A (ConA, 5 µg/mL). The stimulated cells were incubated for 21 h, and then, cell viability was measured by MTT assay.

### Measurement of immunoglobulins, cytokines, and histamine levels by ELISA

Blood samples were collected weekly and the total immunoglobulin E (IgE) levels were measured using an ELISA kit (Bethyl Laboratories, Inc., USA), according to the manufacturer’s instructions. On day 28, the whole blood was obtained and serum was separated by centrifugation. Amounts of IgG2a, histamine, IL-4, and IFN-γ in the serum were measured using a Sandwich ELISA kit (Enzo Life Sciences Inc., Switzerland and BD Biosciences, USA) according to the manufacturer’s instructions. Optical density was measured at 450 nm.

### Histological analysis

The dorsal skin lesions of mice were harvested and immediately fixed in 10% neutral buffered formalin and left for 2 h. The fixed skin was then sliced and embedded in paraffin. Sections were cut from the blocks at 5 µm thickness and stained with hematoxylin and eosin (H&E) for the measurement of skin thickness. Toluidine blue stain was used to analyze the number of mast cells.

### Statistical analysis

Data were expressed as mean ± SD. Student’s *t* test was used to identify statistically significant differences, at a significance level of *p* < 0.05.

## Results

### Anti-oxidant activities of green tea extracts

**Fig. 2:**
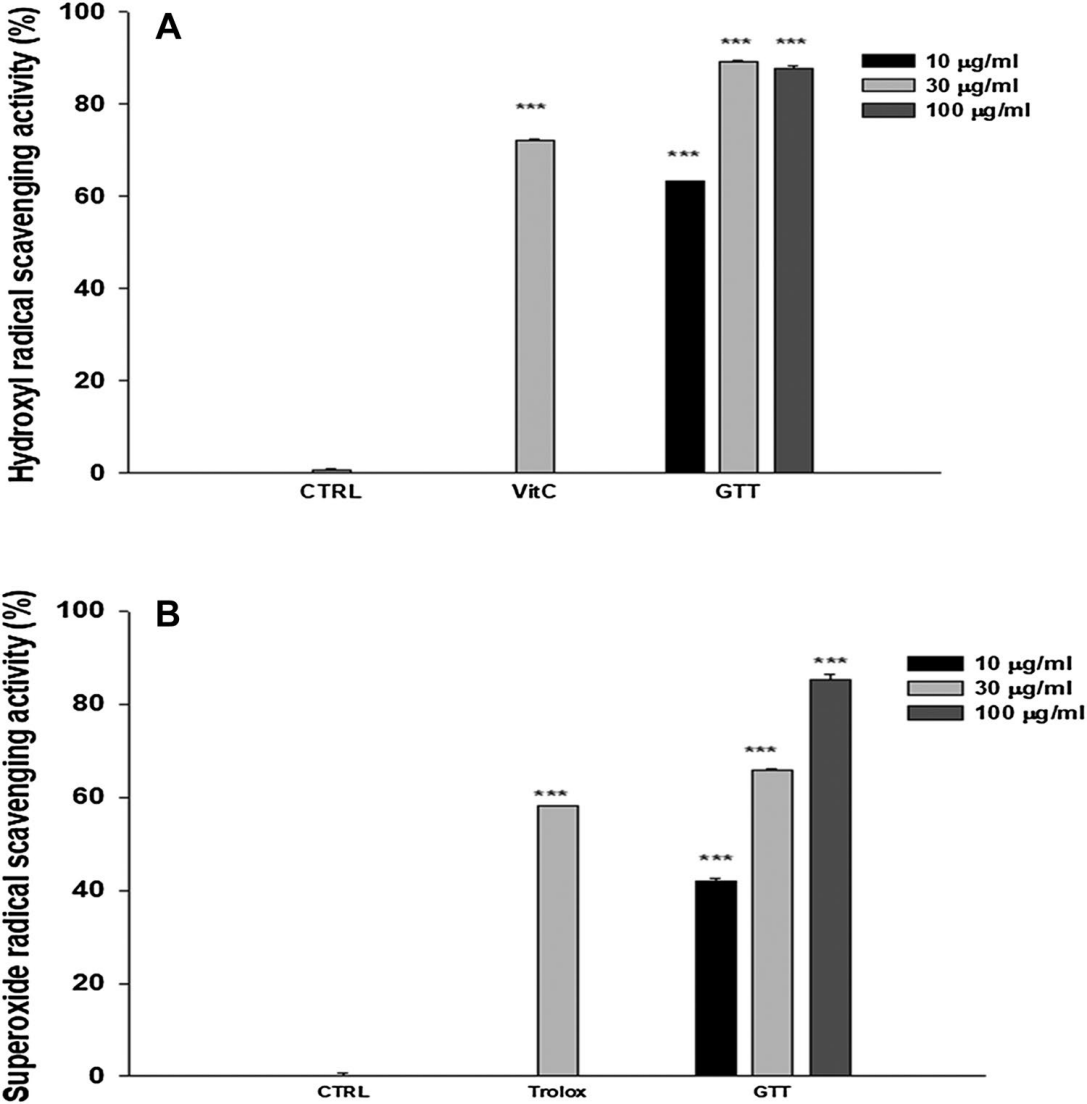
DPPH and SOD radical-scavenging activities of green tea extracts for screening. **a** DPPH radical-scavenging activity increased significantly compared with the control in a dose-dependent manner. **b** Superoxide-radical-scavenging activity was significantly increased compared with the control in a dose-dependent manner. Experiments were performed three times. Data are shown as mean ± SD of the changes in DPPH and superoxide-radical-scavenging activity. ***p < 0.001 compared with the control

The anti-oxidant activities of green tea extracts were determined by measuring the free-radical-scavenging activities for DPPH and SOD. The DPPH free-radical-scavenging activity of green tea extracts increased with increase in the extract concentration. The DPPH radical-scavenging activity of green tea extracts at the concentration range of 10, 30, and 100 µg/mL was 63.2, 89.0, and 87.7%. The positive control, 50 µM of vitamin C, showed the greatest scavenging ability of 72.1%. The scavenging activities of green tea extracts at 30 and 100 µg/mL were significantly higher than the scavenging activity of vitamin C. GTT used in the present investigation was also tested for their superoxide-radical-scavenging activity. The ability to scavenge superoxide radicals ranged from 42.0 to 85.4%. The value increased with an increase in the extract concentrations. The positive control, 500 µg/mL of trolox, had a superoxide-radical-scavenging ability as 58.1%. GTT at concentrations greater than 30 µg/mL was more effective than the positive control. In terms of DPPH and superoxide-radical-scavenging activity, all green tea extracts showed similar anti-oxidative effect (Fig. 2).

### Cytotoxicity of green tea extracts on RBL-2H3 cells

**Fig. 3:**
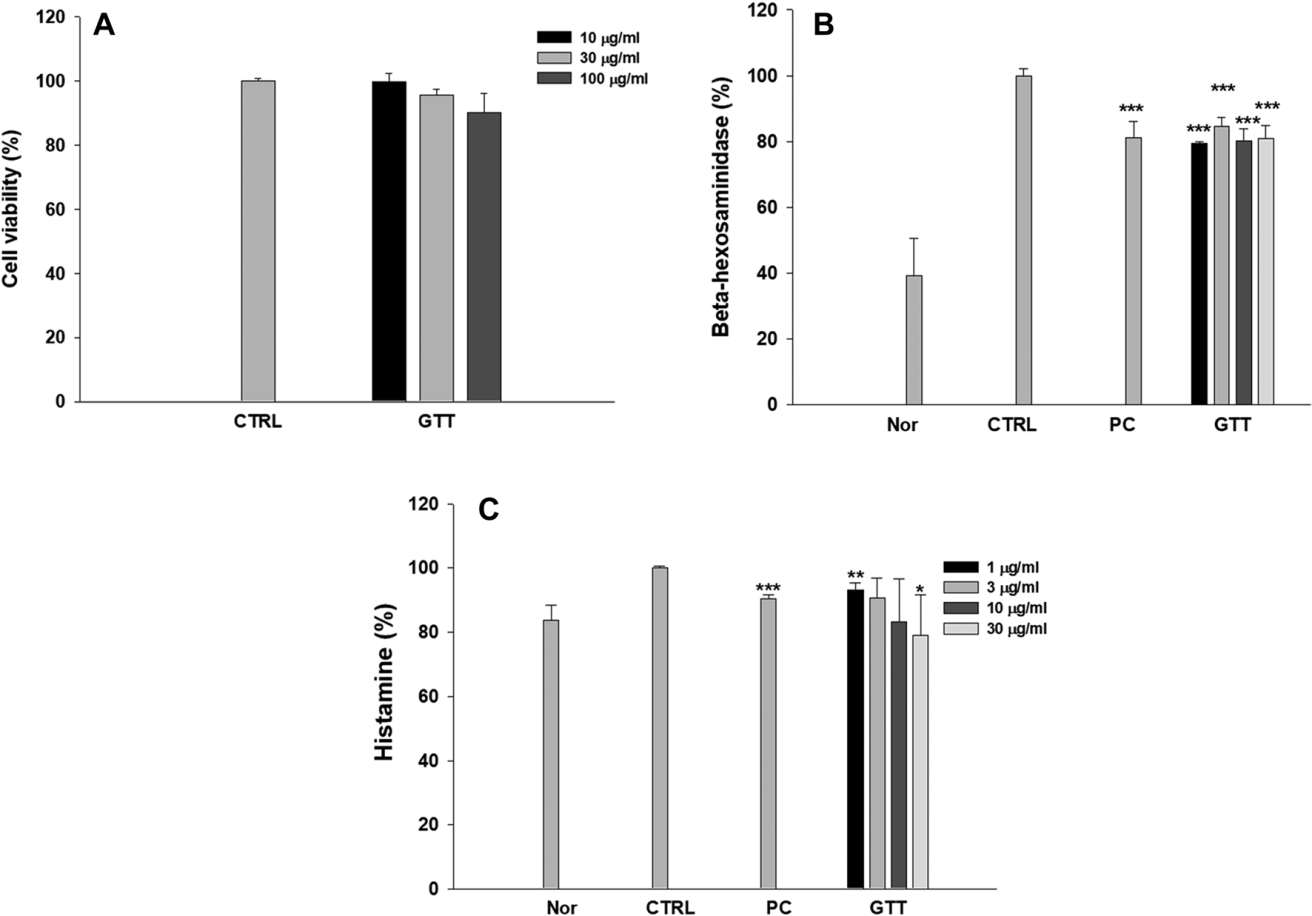
Cytotoxicity of green tea extracts and inhibitory effects on the release of β-hexosaminidase and histamine by green tea extracts on RBL-2H3 cells for screening. **a** Cell viability of green tea extracts on RBL-2H3 cells. Green tea extracts had no significant cytotoxicity on RBL-2H3 cells. **b** Inhibitory effects of green tea extracts on β-hexosaminidase from IgE-antigen complex-stimulated RBL-2H3 cells. Sample no. 7, the green tea GTT, had a significant inhibitory effect at all concentrations. Therefore, GTT was selected for measuring histamine release. **c** Inhibitory effect of GTT on histamine release from IgE-antigen complex-stimulated RBL-2H3 cells. The levels of β-hexosaminidase and histamine were significantly decreased by GTT administration in a dose-dependent manner. Experiments were performed three times. Data are shown as mean ± SD. *p < 0.05, **p < 0.01, and ***p < 0.001 compared with control

To evaluate the cytotoxicity of green tea extracts on RBL-2H3 cells, the cells were treated with different concentrations of green tea extracts for 24 h, and the MTT [3-(4,5-dimethylthiazol-2-yl)-2,5-diphenyl-tetrazolium bromide] method was used for the cytotoxicity assay. As shown in Fig. 3a, green tea extracts had no significant cytotoxicity on RBL-2H3 cells.

### Effect of green tea extracts on the release of β-hexosaminidase and histamine in IgE-antigen complex-stimulated RBL-2H3 cells

The inhibitory effect of green tea extracts on IgE-mediated degranulation in RBL-2H3 cells was measured using β-hexosaminidase and histamine secretions as degranulation biomarkers. The release of β-hexosaminidase and histamine in IgE-antigen complex-stimulated RBL-2H3 cells was significantly increased compared with control cells. Pre-treatment with 1 µM of dexamethasone, the positive control, significantly suppressed the degranulation in IgE-antigen complex-stimulated RBL-2H3 cells. The GTT effectively reduced the release of β-hexosaminidase and histamine at all concentrations in RBL-2H3 cells stimulated with IgE-antigen complex (Fig. 3b, c). Therefore, GTT was chosen for use in AD-like animal model.

### Effect of orally administered GTT on atopic dermatitis (AD) symptoms in NC/Nga mice

To evaluate the therapeutic effect of GTT on AD, AD was induced in mice by topical application of house dust mite (HDM) antigen for 28 days. After 14 days, dermatitis scores increased due to the induced AD, and oral administration of GTT resulted in a dose-dependent reduction in the dermatitis scores. The positive control (5 mg/kg of prednisolone) as well as 400 mg/kg of GTT significantly decreased the dermatitis scores during the experimental period.

When AD gets worse, the skin moisture was decreased. We analyzed the skin moisture content and transepidermal waterloss (TEWL). On day 14, the moisture capacity of the dorsal skin of mice was decreased by the induced AD and prednisolone, and high concentrations of GTT resulted in a significant recovery of the skin moisture. After 21 days, the hair of the dorsal skin interrupted the measurement of skin moisture, so the skin moisture measurements could not be carried out. However, TEWL was measured throughout these experiments, which increased with HDM and decreased by oral administration of prednisolone and GTT. GTT significantly decreased the TEWL in a dose-dependent manner.

**Fig. 4:**
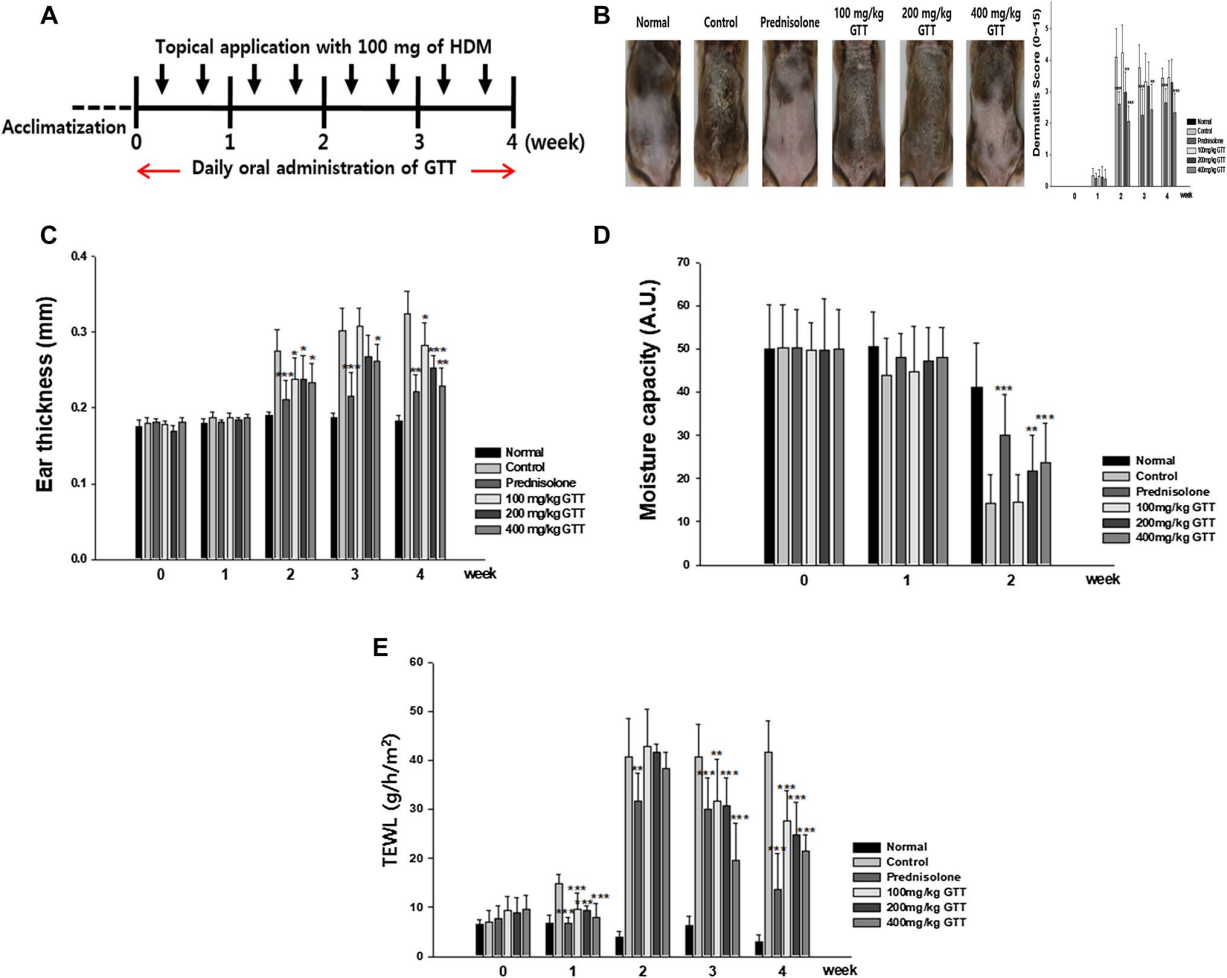
Experimental procedure and changes in the severity of atopic dermatitis-like skin conditions **a** For the induction of AD-like symptoms, house dust mite (HDM) was topically applied to the skin of NC/Nga mice twice weekly for 4 weeks. Mice were fed daily with GTT for 4 weeks. **b** Weekly dermatitis scores were evaluated during the treatment period. **c** Ear thickness was measured with a vernier caliper. **d** Moisture capacity of the skin was measured weekly with a CM825 probe for 2 weeks. **e** Transepidermal waterloss (TEWL) is the evaporation of skin moisture. It was weekly measured with a TM300 probe for 4 weeks. GTT resulted in a significant recovery from atopic dermatitis-like skin condition. Sample size was *n* = 7 per group. Data are shown as mean ± SD. *p < 0.05, **p < 0.01, and ***p < 0.001 compared with the control

As shown in Fig. 4c, the ear thickness became thicker in the AD-induced group. However, oral administration of prednisolone significantly alleviated the thickening of the ears. In addition, GTT significantly alleviated the thickening of the ears in a dose-dependent manner.

### Inhibitory effect of GTT on the proliferation of splenocytes

**Fig. 5:**
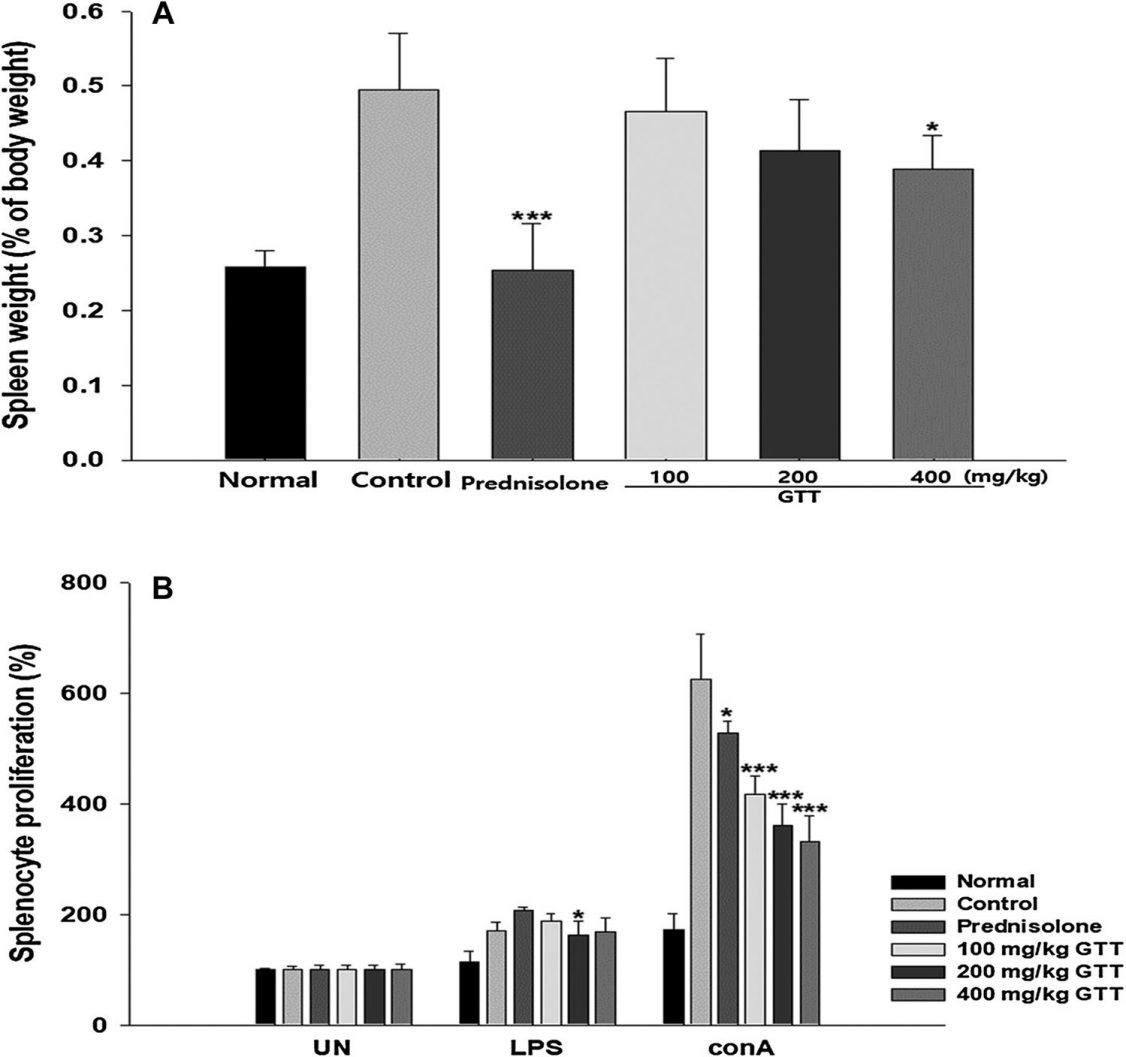
Changes in splenocyte numbers and splenomegaly in atopic dermatitis-like lesions. **a** Spleen weight was measured on day 28. **B** Proliferation of splenocytes obtained from NC/Nga mice was measured by MTT assay on day 28. LPS stimulates B-cell proliferation and ConA stimulates T-cell proliferation. ConA-induced splenocytes were significantly enhanced by the induction of AD. Splenomegaly and the proliferation of T cells were significantly decreased by GTT in a dose-dependent manner. Sample size was *n* = 7 per group. Data are shown as mean ± SD. *p < 0.05 and ***p < 0.001 compared with control

On day 28, we killed the AD-induced mice and the weight of their spleen was measured. Spleen of the AD-induced mice was bigger compared with the control. However, prednisolone and 400 mg/kg GTT treatments resulted in recovered normal weight of the spleen. The inhibitory effect on the proliferation of splenocytes by GTT was determined by MTT assay. LPS stimulates B-cell proliferation and ConA stimulates T-cell proliferation. Both prednisolone and GTT did not affect the LPS-induced splenocyte proliferation, but ConA-induced splenocyte was significantly enhanced by the induced AD and significantly decreased by the administration in a dose-dependent manner. These results suggested that the AD might be related to stimulation of T cells and GTT could alleviate the AD by inhibiting the stimulation of T cells (Fig. 5).

### Effect of GTT on the production of immunoglobulins, cytokines, and histamine

**Fig. 6:**
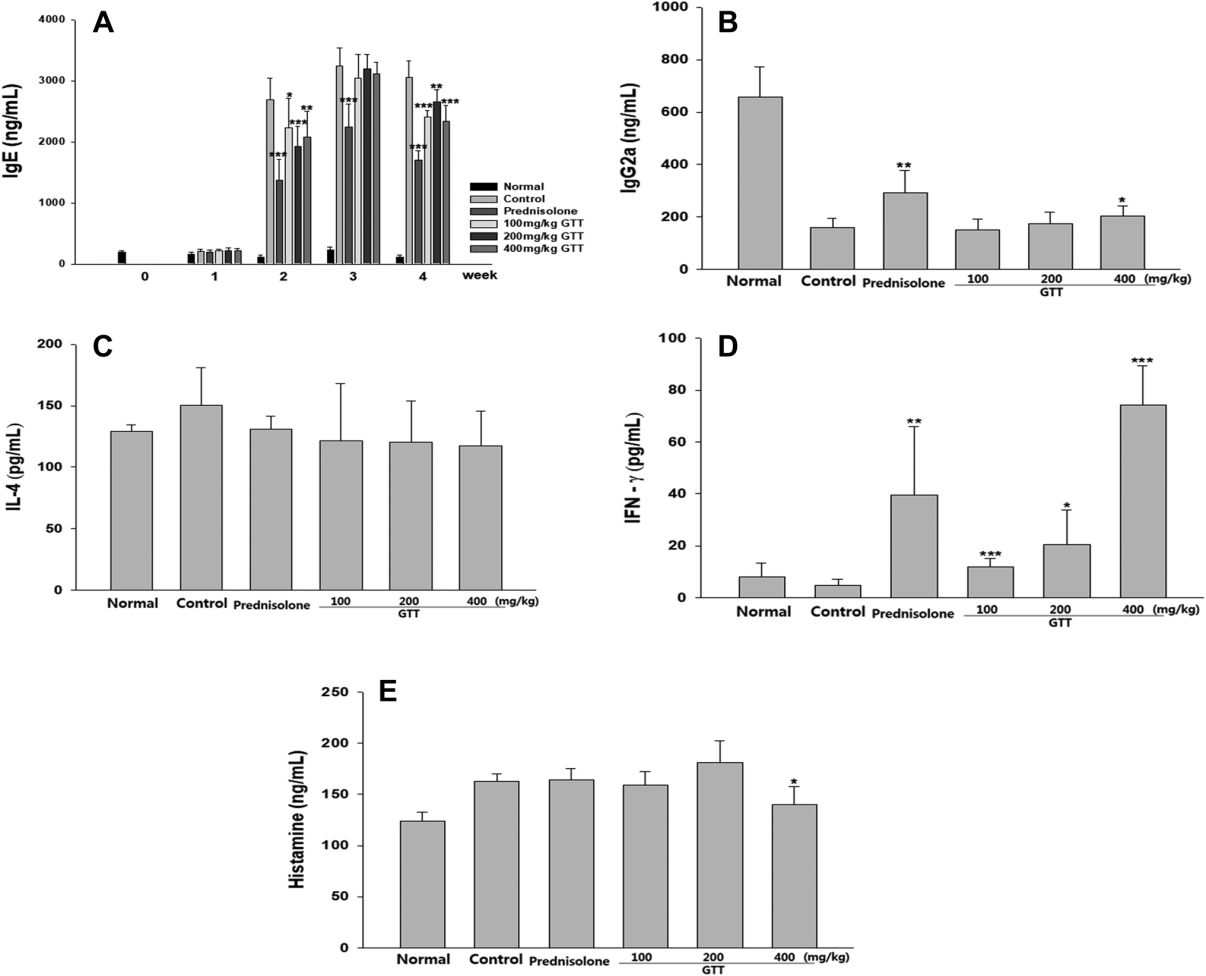
Effect of consecutive administration of GTT on the concentrations of immunoglobulins, cytokines, and histamine in atopic dermatitis-like lesions. **a** Blood samples were collected weekly during the experimental period. On day 28, 400 mg/kg GTT significantly reduced the increased IgE levels in blood. **b** Level of IgG2a was measured using ELISA on day 28. The decreased IgG2a level was increased by the administration of GTT. **c**–**e** The levels of cytokines and histamine in the blood were not significantly changed by the induction of atopic dermatitis. Sample size was *n* = 7 per group. Data are shown as mean ± SD. *p < 0.05, **p < 0.01, and ***p < 0.001 compared with control

To determine whether orally administered GTT affects the production of immunoglobulins, cytokines, and histamine, blood was collected from the mice. Application of HDM antigen resulted in a high concentration of IgE in NC/Nga mice. Compared with the control group, IgE concentration was significantly downregulated in prednisolone and 400 mg/kg GTT-fed groups. Otherwise, IgG2a was decreased by the induced AD and significantly increased by the administration of prednisolone and 400 mg/kg GTT. The amounts of Th1 cytokine IFN-γ and Th2 cytokine IL-4 in the blood did not differ significantly by the induction of AD. The histamine level of blood was significantly increased by the induced AD and decreased by the administration of 400 mg/kg GTT, but there was no inhibitory effect of the positive control, prednisolone (Fig. 6).

### GTT administration decreased skin thickness and local infiltration of mast cells

**Fig. 7:**
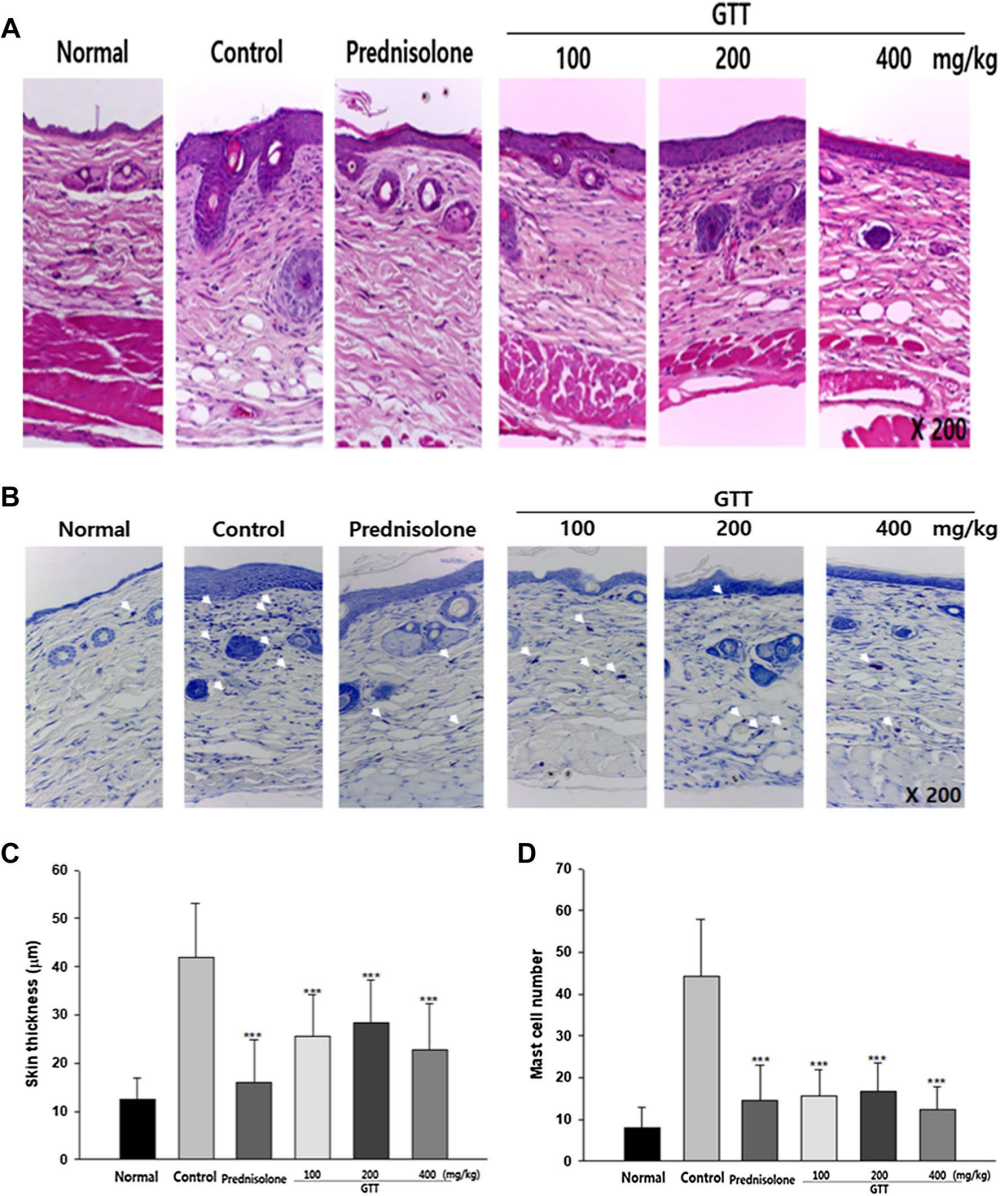
Effect of GTT on skin thickness and local infiltration of mast cells. **a, c** Hematoxylin and eosin stained the dorsal skin sections of NC/Nga mice (× 200). Atopic dermatitis and administration of GTT significantly changed the epidermal thickness. **b, d** Toluidine blue-stained violet mast cells in the skin sections (× 200). GTT inhibited the infiltration of mast cells into the skin. Sample size was *n* = 7 per group. Data are shown as mean ± SD. ***p < 0.001 compared with the control

Figure 7 shows the changes in the histopathological features of the lesions on dorsal skin of NC/Nga mice. The control mice had increased infiltration of inflammatory cells and highly thickened epidermis, with a mean value of 41.9 ± 11.2 µm compared with 12.5 ± 4.3 µm in control mice. The thickness of the epidermis was measured to evaluate the progress of lichenification. Lichenification is a symptom of chronic AD. Prednisolone-fed mice showed recovery in the increased thickness of the skin, showing values similar to the mice without dermatitis. In comparison with the control group, all groups that received GTT showed less hyperplasia in the epidermis and less infiltration of inflammatory cells. Doses of 100, 200, and 400 mg/kg GTT resulted in epidermal thicknesses of 25.5 ± 8.6, 28.4 ± 8.9, and 22.8 ± 9.5 µm, respectively. Infiltrated mast cells in the skin lesions were stained with toluidine blue and counted (Fig. 7b). In contrast to the large numbers of mast cells in the control mice, GTT-fed mice showed significantly fewer mast cells.

## Discussion

Many researchers are constantly studying extraction techniques to improve the functionality and taste of tea [8, 13, 24]. Among them, the role of enzymes in the processing and their application to improve the quality of tea has been recognized. Tea leaves are treated enzymatically with common cell-wall digesting enzymes such as pectinase, cellulose, amylases, and proteases prior to extraction to improve the extract yield and also with tannase to improve cold-water extract ability. Tannase have been characterized mostly by their activity with respect to complex poly phenolics and hydrolyze the ester bond and the depside bond of substrates such as tannic acid, EGCG, ECG, and chlorogenic acid [4]. According to Hong et al., tannase improves the extraction efficiency of polyphenols and increases radical-scavenging activities. In this study, several experiments were performed to evaluate the efficacy of green tea extract in tannase digest (GTT) to in alleviating AD-like immune alteration [14].

In general, AD is an immunological disease resulting from an imbalance favoring type-2 helper T (Th2) cell. Th2 cells, mast cells, or other immune components cells produce interleukin-4 (IL-4), which is known to induce B-cell class switching to IgE [7, 26, 29]. Up-regulation of IgE production is a hallmark of AD in humans [15]. Th1 cells, natural killer cells, and other immune cells produce IFN-γ that triggers the isotype-switching to IgG2a in mice [31, 32]. Therefore, analysis of the IgE and IgG2a levels could reflect the alteration of homeostasis between type-1 and type-2 immune responses.

Likewise, mast cells are the primary effector cells involved in the allergic inflammatory reaction. The degranulation process starts by the interaction of antigens with mast cells [37]. In addition, mast cells secrete a variety of bioactive substances such as histamine and pro-inflammatory cytokines [22, 25]. Histamine is a hallmark of allergic responses and induces vascular permeability [2]. Since histamine is immediately released following exposure to allergen, an increased histamine level is often considered a parameter to diagnose the onset of allergic diseases [16]. This study initially screened several green tea extracts based on their anti-oxidant ability and the inhibitory effect due to the release of β-hexosaminidase on IgE-antigen complex-stimulated RBL-2H3 cells. The selected green tea extract also inhibited the release of histamine in the same cells. RBL-2H3 is a basophilic leukemia cell line and has characteristics of mucosal-type mast cells [6]. This cell line is considered to be a good model for studying comprehensive events on mast cell activation [28]. GTT significantly suppressed the release of β-hexosaminidase and histamine without cytotoxic effect on mast cells. As a result, we selected GTT as the therapeutic ingredient for AD.

The NC/Nga mouse has a mutation on chromosome 9, which is related to the increased production of IgE and a Th2-dominant inflammation [1]. However, exposure to a standard environment alone is not enough to reveal AD-like symptoms in these mice. Continuous skin irritation with certain environmental allergens (e.g., house dust mite allergens) in NC/Nga mice could provoke AD-like symptoms [19]. In this study, AD was induced twice a week for 28 days and GTT was orally administered daily at the same periods. The dermatitis scores were evaluated by observing erythema, dry skin, edema/excoriation, erosion, and lichenification [36]. The skin thickness was used as an indicator of lichenification, frequently observed in chronic AD [38]. The ears thickened by AD were thinned by GTT. Administration of GTT increased the moisture capacity of the skin and reduced the moisture evaporation of the skin, thereby alleviating the dryness of the skin. These results showed that the administration of GTT effectively regulates various clinical symptoms associated with AD.

Atopic dermatitis is an inappropriate immune response, involving an excessive Th2 response [5]. This study showed that the proliferation of T-cell stimulation by con A was increased by the induced AD and decreased by the administration of GTT. The type-2 response becomes predominant when the differentiation or activation of Th2 cells is preferred, but development or stimulation of Th1 cells is suppressed [12, 27]. Orally administered GTT decreased the IgE and increased the IgG2a in the blood of NC/Nga mice. It suggests a possibility that GTT is able to modulate the immune activation of mast cells, because these cells have the surface receptor for IgE to induce their activation and secretion of inflammatory mediators [34]. The epidermal thickness in GTT-administered mice was thinned compared with the mice in the control group. The infiltration of various immune cells, including mast cells, into the skin lesions is a characteristic feature of AD [17]. Mast cells play a central role in AD through degranulation of inflammatory mediators by binding IgE onto the cells [21]. Decreased infiltration of mast cells by GTT administration is expected to play a role in the reduction of skin inflammation.

In conclusion, orally administered green tea extract from tannase digest (GTT) suppressed the inflammation of AD-like skin lesions, and decreased the immune cell infiltration into the skin. These results suggest that GTT may have a therapeutic potential in the treatment of AD.
